# Puf4 Mediates Post-transcriptional Regulation of Cell Wall Biosynthesis and Caspofungin Resistance in Cryptococcus neoformans

**DOI:** 10.1128/mBio.03225-20

**Published:** 2021-01-12

**Authors:** Murat C. Kalem, Harini Subbiah, Jay Leipheimer, Virginia E. Glazier, John C. Panepinto

**Affiliations:** a Department of Microbiology and Immunology, Witebsky Center for Microbial Pathogenesis and Immunology, Jacobs School of Medicine and Biomedical Sciences, University at Buffalo, SUNY, Buffalo, New York, USA; b Department of Biology, Niagara University, Niagara, New York, USA; Duke University Medical Center

**Keywords:** antifungal resistance, caspofungin, cell wall, post-transcriptional, RNA-binding proteins

## Abstract

The human fungal pathogen Cryptococcus neoformans is intrinsically resistant to the echinocandin antifungal drug caspofungin, which targets the β-1,3-glucan synthase encoded by *FKS1*. Echinocandins have been on the market for 20 years, yet they are the newest class of antifungal drugs. Analysis of a C. neoformans *puf4*Δ mutant, lacking the pumilio/FBF RNA binding protein family member Puf4, revealed exacerbated caspofungin resistance. In contrast, overexpression of *PUF4* resulted in caspofungin sensitivity. The *FKS1* mRNA contains three Puf4-binding elements (PBEs) in its 5′ untranslated region. Puf4 binds with specificity to this region of *FKS1*. The *FKS1* mRNA was destabilized in the *puf4*Δ mutant, and the abundance of the *FKS1* mRNA was reduced compared to wild type, suggesting that Puf4 is a positive regulator of *FKS1* mRNA stability. In addition to *FKS1*, the abundance of additional cell wall biosynthesis genes, including chitin synthases (*CHS3*, *CHS4*, and *CHS6*) and deacetylases (*CDA1*, *CDA2*, and *CDA3*) as well as a β-1,6-glucan synthase gene (*SKN1*), was regulated by Puf4. The use of fluorescent dyes to quantify cell wall components revealed that the *puf4*Δ mutant had increased chitin content, suggesting a cell wall composition that is less reliant on β-1,3-glucan. Overall, our findings suggest a mechanism by which caspofungin resistance, and more broadly, cell wall biogenesis, is regulated post-transcriptionally by Puf4.

## Abstract (precis)

Cryptococcus neoformans is an environmental fungus that causes pulmonary and central nervous system infections. It is also responsible for 15% of AIDS-related deaths.

## INTRODUCTION

Invasive deep mycoses primarily impact immunocompromised populations, causing high rates of morbidity and mortality (1, 2). The pathogenic fungus Cryptococcus neoformans is the causative agent of fatal meningitis most often in patients with defects in cell-mediated immunity, including transplant recipients and those living with HIV/AIDS (3–6). C. neoformans is responsible for 15% of AIDS-related deaths (6). Treatment of cryptococcosis is difficult, and therapeutic options are limited. Even the best combination treatment using amphotericin B with 5-fluorocytosine (5-FC) is not well tolerated, and 5-FC is often unavailable in resource-poor areas (7). Some of the most considerable clinical challenges of invasive fungal infections are low efficacy of drugs, emerging resistance issues, and limited variety and availability of antifungals, especially in the areas where they are needed the most (8, 9).

Another antifungal agent, fluconazole, is largely ineffective as first-line therapy. It lacks effective fungicidal activity against C. neoformans *in vivo* even at high concentrations and presents resistance issues (10, 11). Echinocandins (such as caspofungin, micafungin, and anidulafungin) are the latest class of antifungal drugs approved by the Food and Drug Administration (FDA) that target cell wall biosynthesis. The echinocandins are ineffective against C. neoformans due to a high level of intrinsic resistance (12). Echinocandins specifically target the β-1,3-glucan synthase encoded by *FKS1*, and C. neoformans Fks1 is sensitive to inhibition by the echinocandins, suggesting that the mechanism of intrinsic resistance is not related to biochemical differences in the target itself (13). In other pathogenic fungi, such as *Aspergillus* and *Candida* species, resistance to caspofungin is manifested due to mutations in *FKS1*, but this is not observed in *Cryptococcus* (14, 15). Another mechanism that is discussed regarding caspofungin resistance in pathogenic fungi involves the cell wall remodeling and integrity pathways (14–16). It has been shown that increased cell wall chitin content contributes to caspofungin resistance (17, 18). Additionally, a defect in intracellular drug concentration maintenance due to drug influx and efflux imbalance has been proposed to be a potential mechanism to explain the intrinsic resistance phenomenon (19, 20). Discovering and targeting the regulatory components behind the pathways involved in the intrinsic resistance may result in a combination therapy that potentiates the antifungal activity of caspofungin toward C. neoformans.

Calcineurin signaling plays a distinct role in intrinsic caspofungin resistance (21, 22). Caspofungin synergizes with the calcineurin inhibitors FK506 and cyclosporine (23). Both the A and B subunits of calcineurin regulate tolerance to caspofungin (19). Crz1, the transcription factor that is activated through dephosphorylation by calcineurin, translocates to the nucleus following treatment with caspofungin, yet the *crz1*Δ mutant exhibits no sensitivity or resistance to the drug, suggesting caspofungin resistance is Crz1 independent. (19). Calcineurin functions at the intersection of multiple signaling pathways and interacts with a diversity of proteins involved in calcium signaling, RNA processing, protein synthesis, and vesicular trafficking, among others (24, 25). Since caspofungin resistance is calcineurin dependent, yet Crz1 independent, RNA processing targets of calcineurin that may be involved in resistance to caspofungin through post-transcriptional modulation of gene expression are especially of interest. Post-transcriptional gene regulation in drug resistance phenotypes is largely unexplored in fungal pathogens, and investigation of post-transcriptional regulatory networks may identify targets for adjunctive therapies to improve the efficacy of drugs.

One of the targets of calcineurin involved in RNA processing is a pumilio-domain and FBF (PUF) domain-containing RNA-binding protein, Puf4 (24). C. neoformans Puf4 is homologous to the Saccharomyces cerevisiae paralogs Puf4 and Mpt5 (26). Our previous work demonstrated that C. neoformans Puf4 recognizes the Mpt5 binding element in its mRNA targets (27). It has been hypothesized that Puf4 may play a role in the regulation of cell wall biosynthesis since the *puf4*Δ mutant is resistant to lysing enzymes, is temperature sensitive at both 37°C and 39°C, and is sensitive to Congo red (28). Our group has previously shown that Puf4 regulates endoplasmic reticulum (ER) stress through controlling the splicing of a major ER stress-related transcript, *HXL1*, and plays a role in the unfolded protein response pathway of C. neoformans. Puf4 also contributes to virulence, as the *puf4*Δ mutant has attenuated virulence compared to wild type in an intravenous murine competition model of cryptococcosis (27).

In this study, we report that Puf4 contributes to the intrinsic caspofungin resistance of C. neoformans through post-transcriptional regulation of the mRNA encoding the drug target, Fks1. Puf4 also regulates a number of the cell wall biosynthesis-related mRNAs. This regulation is primarily at the level of mRNA stability and has functional consequences in maintaining cell wall composition.

## RESULTS

### The *puf4*Δ mutant is resistant to caspofungin.

**FIG 1:**
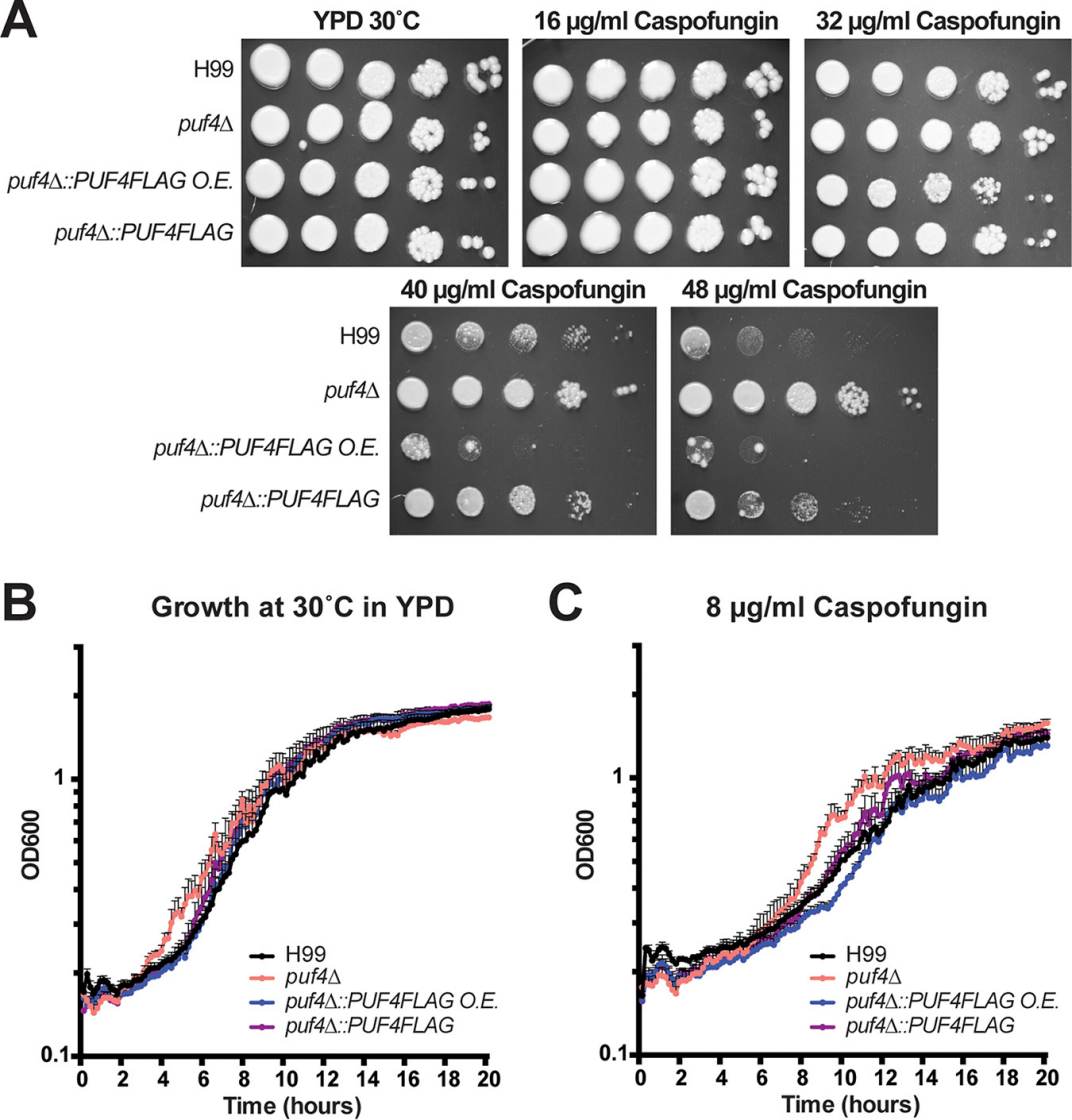
The *puf4*Δ mutant is resistant to caspofungin. (A) Spot plate analysis. The indicated strains were diluted to an OD_600_ of 1.0, and five 1:10 serial dilutions were spotted on agar plates containing 0, 16, 32, 40, and 48 µg/ml caspofungin. Plates were incubated at 30°C for 3 days and photographed. (B and C) Growth assay. The indicated strains were diluted to an OD_600_ of 0.3 and mixed 1:1 with either fresh YPD or YPD containing caspofungin in a 96-well plate. Plates were incubated at 30°C for 20 h while shaking. OD_600_ was measured every 10 min.

Our previous work has implicated the pumilio family RNA binding protein Puf4 in the regulation of ER stress in C. neoformans (27). Puf4 is an effector of calcineurin signaling, a pathway known to regulate thermotolerance and cell integrity (24, 28). Given the connection of Puf4 to cell integrity signaling, we assessed the sensitivity of the *puf4*Δ mutant to the cell wall-perturbing drug caspofungin. We measured caspofungin sensitivity by spot plate analyses and found that the *puf4*Δ mutant is resistant to caspofungin above the published MIC of 16 µg/ml (Fig. 1A). This result suggests that Puf4 is a negative regulator of caspofungin resistance in C. neoformans since its absence presents a hyperresistant phenotype. When Puf4 was overexpressed with a FLAG tag (3 copies determined by Southern blotting), not only was the hyperresistance suppressed, but the strain was more sensitive to caspofungin. Single-copy Puf4-FLAG complementation of the *puf4*Δ mutant restored a wild-type resistance phenotype (Fig. 1A). In addition to the spot plate analyses, growth analysis using liquid cultures in a kinetic plate reader assay in the presence of 8 µg/ml caspofungin showed trends similar to the phenotypes observed in spot plates (Fig. 1B). Growth was not permissible in the presence of 16 µg/ml caspofungin in liquid culture for both wild-type and mutant cells, suggesting that the action of caspofungin may be influenced by the environmental constraints under different culture conditions (data not shown).

### *PUF4* transcript and protein expression are decreased following caspofungin treatment.

**FIG 2:**
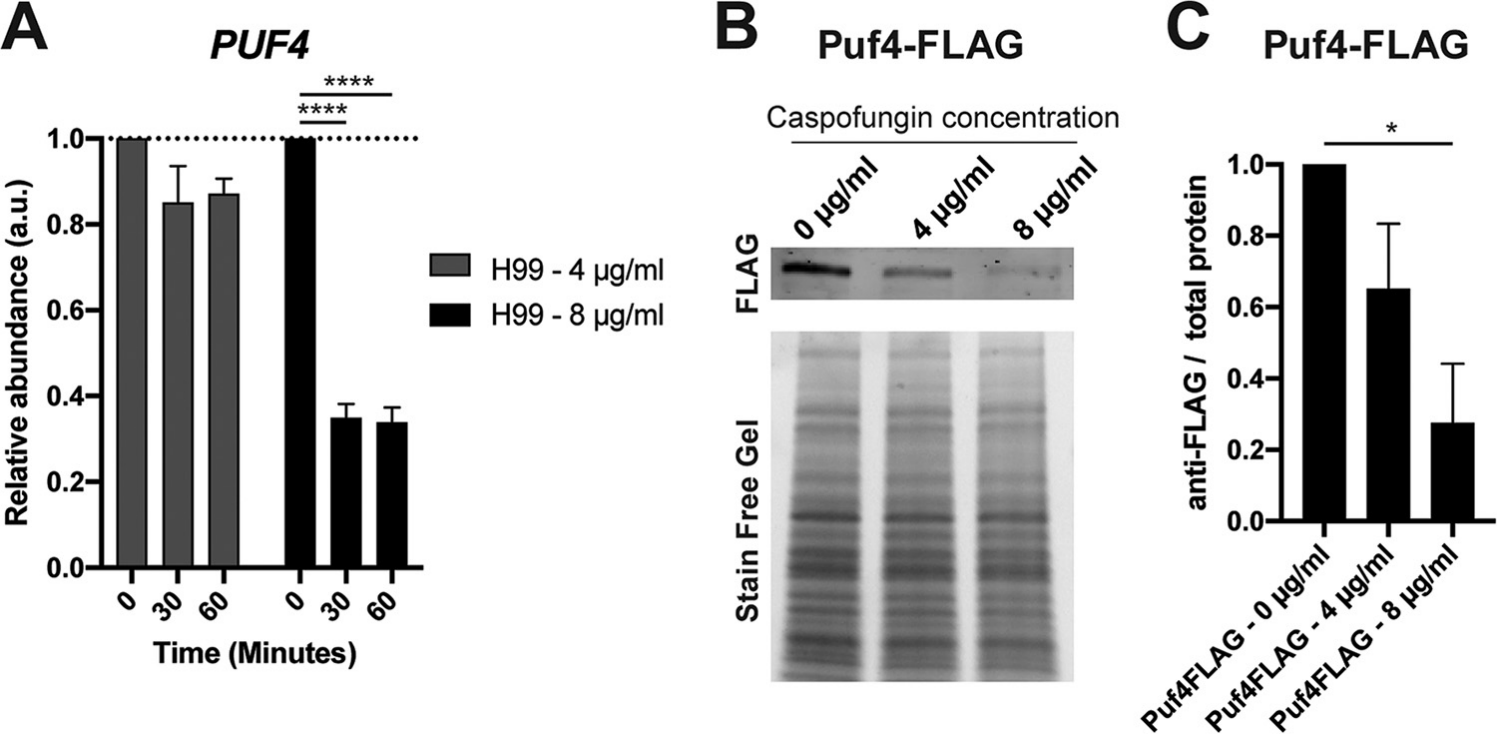
Puf4 transcript and protein levels are decreased following caspofungin treatment. (A) *PUF4* transcript levels are decreased during a 60-min caspofungin time course. Cells were grown at 30°C and treated with 4 or 8 µg/ml caspofungin. The abundance of *PUF4* mRNA was determined in samples collected at 30 and 60 min after caspofungin treatment using RT-qPCR with *GPD1* as the normalization gene. Three replicates were plotted, and one-way-ANOVA with Tukey’s test was performed to assess statistical significance. (B) Puf4 protein levels are decreased at 60 min after caspofungin treatment. The Puf4-FLAG strain (*puf4*Δ expressing *PUF4-FLAG* C-terminal fusion protein) was grown to mid-log phase and treated with caspofungin for 1 h. A representative SDS-PAGE assay followed by immunoblotting using anti-FLAG antibody is shown. (C) Anti-FLAG signal is normalized to total protein signal from the stain-free gel. Three replicates were plotted, and unpaired *t* test with Welch’s correction was performed. *, *P* < 0.05.

To understand the involvement of Puf4 in the caspofungin resistance phenotype, we first set out to determine the *PUF4* transcript and protein levels during caspofungin treatment. We performed a caspofungin time course in which we treated wild-type cultures grown to mid-log phase with either 4 or 8 µg/ml caspofungin for 30 or 60 min. Caspofungin concentrations were picked based on viability analysis (see Fig. S1 in the supplemental material). We quantified the *PUF4* transcript levels and found out that the *PUF4* transcript levels are decreased following treatment with 8 µg/ml caspofungin (Fig. 2A). Lower caspofungin concentration did not significantly influence the PUF4 transcript levels. To evaluate the Puf4 protein levels, we grew Puf4-FLAG cells to the mid-log stage and treated them with 4 or 8 µg/ml caspofungin for 60 min. We detected the Puf4-FLAG using immunoblotting with an anti-FLAG antibody. We found that Puf4 protein levels are downregulated following caspofungin treatment. Both 4- and 8-µg/ml concentrations caused downregulation, ∼40% and ∼80%, respectively (Fig. 2B and C). Since the absence of Puf4 causes a hyperresistance phenotype, we speculate that the downregulation of Puf4 protein abundance following caspofungin treatment may be a contributing event to the intrinsic resistance to caspofungin. This lays emphasis on the importance of understanding the genetic interactions of players involved in post-transcriptional gene regulatory networks that are not known as direct drug targets yet influence the drug resistance phenotypes.

10.1128/mBio.03225-20.1 FIG S1 Caspofungin is a fungicidal drug for Cryptococcus neoformans. H99 and *puf4*Δ cells were grown to mid-log phase and treated with 4 or 8 µg/ml caspofungin. Cells were harvested following a 1-h incubation, and multiple serial dilutions were plated on YPD agar to assess survival. YPD agar plates were incubated at 30°C for 2 days, and colonies were counted using a protoCOL3 colony counter. Data presented as percent CFU relative to untreated. Download FIG S1, TIF file, 1.7 MB. Copyright © 2021 Kalem et al. 2021 Kalem et al. This content is distributed under the terms of the Creative Commons Attribution 4.0 International license.

### Puf4 directly binds and stabilizes the *FKS1* mRNA.

**FIG 3:**
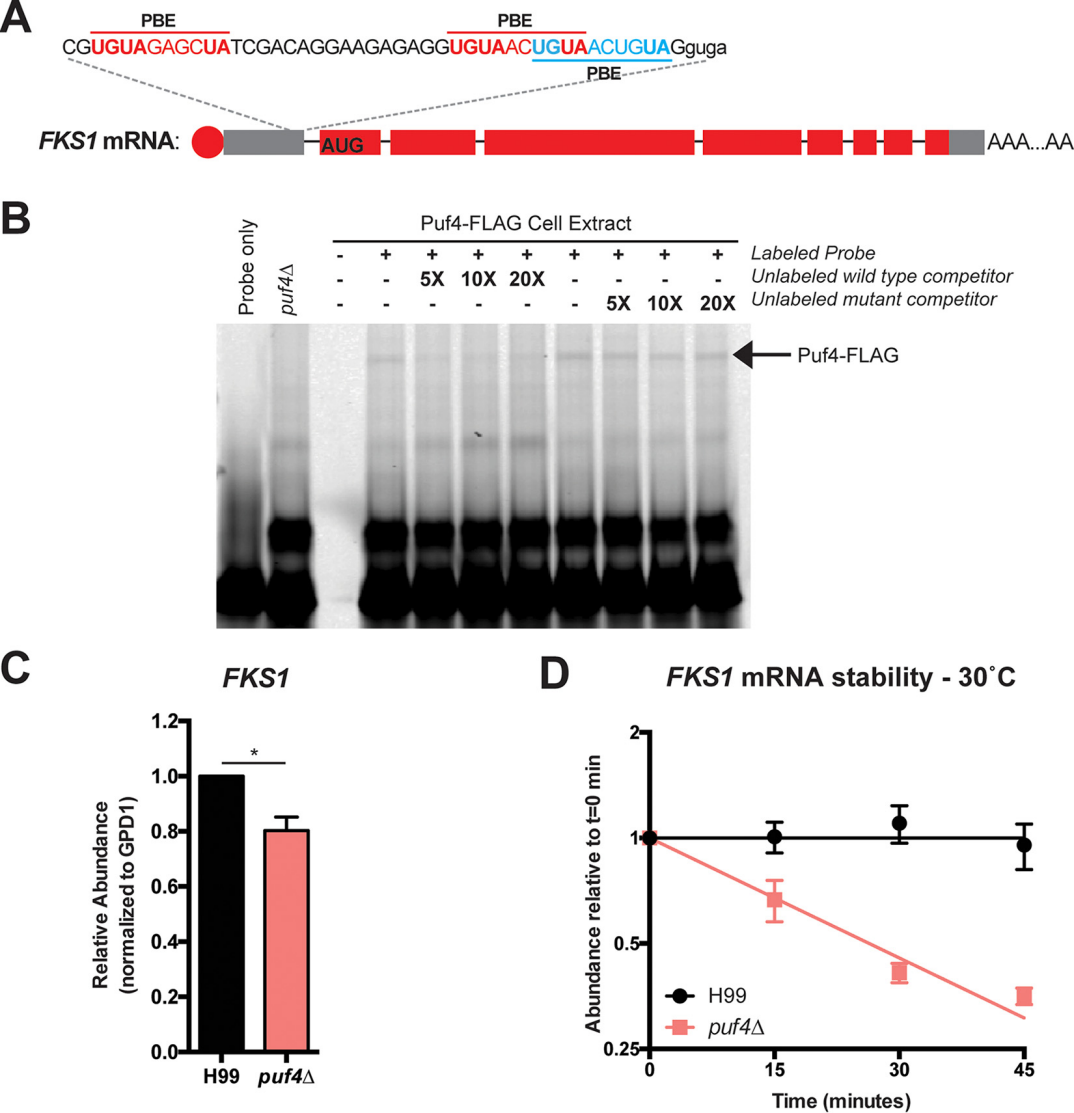
Puf4 directly binds and stabilizes the *FKS1* mRNA. (A) The *FKS1* mRNA contains Puf4 binding elements (PBEs) in its 5′ UTR, UGUANNNNUA. (B) Puf4 binds to *FKS1* mRNA. Electrophoretic mobility shift assay was performed using a fluorescently labeled synthetic RNA oligonucleotide designed for the *FKS1* 5′ UTR that contain the PBEs. Unlabeled mutant (containing mutated PBEs) and unlabeled wild-type probes were used as controls for sequence specificity. The *puf4*Δ mutant was included as a control to show that binding is absent when Puf4 is not present. A representative gel image is shown (*n* = 3). (C) The *FKS1* mRNA is downregulated in the *puf4*Δ mutant. The *FKS1* mRNA abundance in mid-log samples grown at 30°C was determined using RT-qPCR with *GPD1* as the normalization gene. Three replicates were plotted, and unpaired *t* test with Welch’s correction was performed. *, *P* < 0.05. (D) The *FKS1* mRNA is destabilized in the *puf4*Δ mutant. *FKS1* abundance was determined using RT-qPCR following transcription shutoff using 1,10-phenanthroline to determine the mRNA decay kinetics. *GPD1* was utilized as the control for normalization. Three replicates were plotted, and differences between two strains were analyzed using one-phase exponential decay analysis.

The target of caspofungin, β-1,3-glucan synthase, is encoded by the *FKS1* gene. We searched the *FKS1* mRNA sequence for a potential Puf4 binding element (PBE). Our previous work suggests that the Puf4 binding element in C. neoformans is homologous to that of S. cerevisiae Mpt5, including the invariant initiating UGUA followed by a 4-nucleotide spacer sequence and terminating UA. We found that the *FKS1* mRNA contains three Puf4 binding elements in its 5′ untranslated region (UTR) (Fig. 3A). To determine if Puf4 can directly interact with its consensus elements in the 5′ UTR of the *FKS1* mRNA, we performed an electrophoretic mobility shift assay (EMSA). We synthesized a 50-base-long fluorescently labeled (TYE705 infrared label) RNA oligonucleotide that spans the *FKS1* 5′ UTR containing the all three Puf4-binding elements (see Table S1 in the supplemental material). Incubation of the fluorescent oligonucleotide with the wild-type cell lysate resulted a shift that was competed with the unlabeled oligonucleotide. A mutant unlabeled competitor, in which the UGUANNNNUA motif was replaced by adenines, was unable to compete for binding with the wild-type fluorescent oligonucleotide. This suggests that Puf4 binds to the *FKS1* mRNA through sequence-specific recognition of the Puf4 binding elements in the 5′ UTR (Fig. 3B). Incubation of the fluorescent oligonucleotide with the *puf4*Δ mutant lysate shows the absence of a shift that was observed with the Puf4-FLAG cell lysate (Fig. 3B, arrow). Because we detected an interaction between Puf4 and the 5′ UTR sequence of *FKS1*, we went on to investigate if loss of Puf4 would alter the abundance or stability of *FKS1* mRNA. *FKS1* mRNA abundance in mid-log-grown cultures of the *puf4*Δ mutant was decreased 20% compared to wild-type cells grown in parallel (Fig. 3C). Puf proteins are known mRNA stability regulators, and so we asked if the reduction in *FKS1* mRNA in the *puf4*Δ mutant was due to destabilization. We performed an mRNA stability time course following transcription shutoff using 1,10-phenanthroline and found that the *FKS1* mRNA was destabilized in the absence of Puf4 compared to the wild type (Fig. 3D).

10.1128/mBio.03225-20.3 TABLE S1 Primers used in this study. List of primers and oligonucleotides used in cloning, RT-qPCR, and EMSA experiments. Download Table S1, XLSX file, 0.09 MB. Copyright © 2021 Kalem et al. 2021 Kalem et al. This content is distributed under the terms of the Creative Commons Attribution 4.0 International license.

### Lack of Puf4-dependent *FKS1* regulation leads to increased Fks1 protein abundance in the *puf4*Δ mutant.

**FIG 4:**
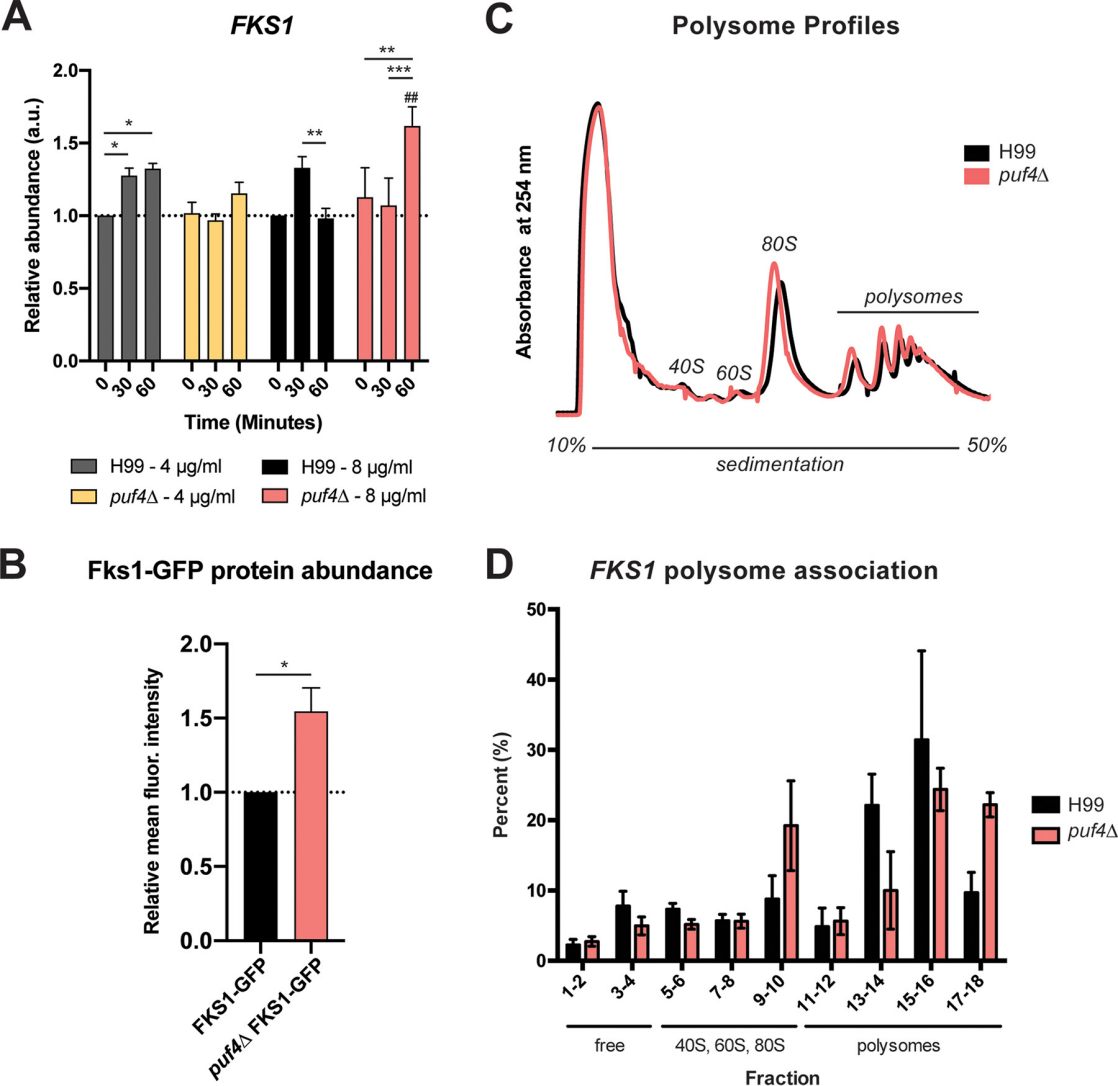
Puf4 controls the *FKS1* abundance during caspofungin treatment and negatively regulates *FKS1* translation. (A) *FKS1* mRNA abundance is regulated by Puf4 during a 60-minute caspofungin time course. Cells were grown at 30°C and treated with 4 and 8 µg/ml caspofungin. The abundance of *FKS1* mRNA was determined in samples collected at 30 and 60 min after caspofungin treatment using RT-qPCR with *GPD1* as the normalization gene. Three replicates were plotted, and one-way ANOVA with Tukey’s test was performed. (B) Puf4 is a negative regulator of Fks1 protein abundance. FKS1-GFP and *puf4*ΔFKS1-GFP cell lines were grown in tissue culture medium, and GFP levels were analyzed using flow cytometry. Mean fluorescence intensities are plotted relative to FKS1-GFP cell line, and an unpaired *t* test with Welch’s correction was performed. *, *P* < 0.05. (C and D) Puf4 negatively regulates the translation of *FKS1*. Wild-type and the *puf4*Δ mutant cell lysates were analyzed using polysome profiling. No gross differences in polysome traces were observed among the strains (C). RNA was extracted from polysome fractions to determine the abundance of the *FKS1* mRNA in fractions corresponding to free RNAs, 40S and 60S ribosomal subunits, 80S monosome, and polysomes. Percent distribution of the FKS1 mRNA across the gradient is graphed for both strains.

We next investigated the *FKS1* mRNA levels following treatment with 4 or 8 µg/ml caspofungin to determine if inhibition of the enzyme activates a feedback mechanism to upregulate expression of *FKS1* mRNA. The *FKS1* mRNA abundance is upregulated in wild-type cells at 30 and 60 min following treatment with 4 µg/ml caspofungin compared to 0 min (Fig. 4A). Conversely, the *puf4*Δ mutant cells have no change in *FKS1* mRNA abundance at 30 min after caspofungin treatment (Fig. 4A). *FKS1* mRNA abundance is significantly upregulated at the 60-min time point when cells are treated with 8 µg/ml caspofungin compared to the zero time point. This upregulation is absent in wild-type cells. These different trends in transcript abundance following caspofungin treatment may suggest that Puf4 may play a role in modulating Fks1 protein expression and consequently the cell wall β-1,3-glucan levels.

We reasoned that if Puf4 is regulating *FKS1* mRNA abundance and stability, it may also contribute in regulating Fks1 protein expression. We utilized an *FKS1*-GFP (green fluorescent protein) parent cell line that had an in-locus GFP tag (29), and generated a *puf4*Δ-*FKS1*-GFP cell line. We analyzed the GFP fluorescent signal in both cell lines using flow cytometry as a direct way to measure Fks1 abundance in the cell. Representative microscopy images are included in Fig. S2. We observed that the *puf4*Δ cells had an ∼50% increase in Fks1 protein abundance (Fig. 4B). In accordance with mRNA stability data, this finding supports a mechanism in which Puf4 stabilizes and negatively regulates the translation of Fks1 mRNA. To further scrutinize this mechanistic scenario, we performed sucrose gradient polysome fractionations of both wild-type and *puf4*Δ cells. We pooled every other sucrose gradient fraction, extracted RNA, and measured the abundance of *FKS1* mRNA in each pool. Results showed that the *puf4*Δ mutant had more *FKS1* mRNA present in the pools corresponding to 80S monosome (single ribosomes) and heavy polysome (multiple actively translating ribosomes) fractions (Fig. 4C and D). This suggests that the increase in Fks1 protein abundance in the *puf4*Δ mutant is due to increased translation of the *FKS1* mRNA, consistent with a role for Puf4 in repressing the translation of *FKS1* mRNA.

10.1128/mBio.03225-20.2 FIG S2 Fks1-GFP expression is upregulated in the *puf4*Δ cells. Cells were grown overnight in tissue culture medium (RPMI). GFP fluorescent signal was detected using a Leica TCS SP8 confocal microscope. Representative GFP and DIC images are shown. Download FIG S2, TIF file, 2.0 MB. Copyright © 2021 Kalem et al. 2021 Kalem et al. This content is distributed under the terms of the Creative Commons Attribution 4.0 International license.

### Puf4 binds and regulates the expression of cell wall biosynthesis genes other than *FKS1*.

A previous study revealed that multiple cell wall genes are influenced by caspofungin treatment (19). We assessed cell wall biosynthesis mRNAs for the presence of a putative Puf4 binding element and found that several caspofungin-sensitive genes contain Puf4 binding elements (Table 1). These include genes that encode chitin synthases, chitin deacetylases, and α-glucan and β-glucan synthases.

**TABLE 1:** List of Puf4 binding element-containing cell wall biosynthesis-related genes^*a*^

Gene	Gene ID	Start	End	*P* value	Location
CHS3*	CNAG_05581	242	251	0.000262	5′ UTR
CHS4*	CNAG_00546	3257	3266	0.00013	Exon
		3516	3525	0.000203	Exon
*CHS7*	CNAG_02217	88	97	6.59E−05	5′ UTR
		3264	3273	6.59E−05	Exon
*CHS8*	CNAG_07499	1479	1488	7.96E−05	Exon
CDA3*	CNAG_01239	1916	1925	7.96E−05	3′ UTR
FKS1*	CNAG_06508	281	290	7.96E−05	5′ UTR
		307	316	0.000162	5′ UTR
		313	322	0.000162	5′ UTR
*SKN1*	CNAG_00897	2471	2480	0.000122	3′ UTR
AGS1*	CNAG_03120	202	211	0.000107	5′ UTR

a The UGUANNNNUA motif was searched in target genes using FIMO (MEME-suite version 5.1.1.). Results were manually confirmed, and locations of the motifs were identified. Asterisks indicate the genes selected for further mRNA stability analysis.

**FIG 5:**
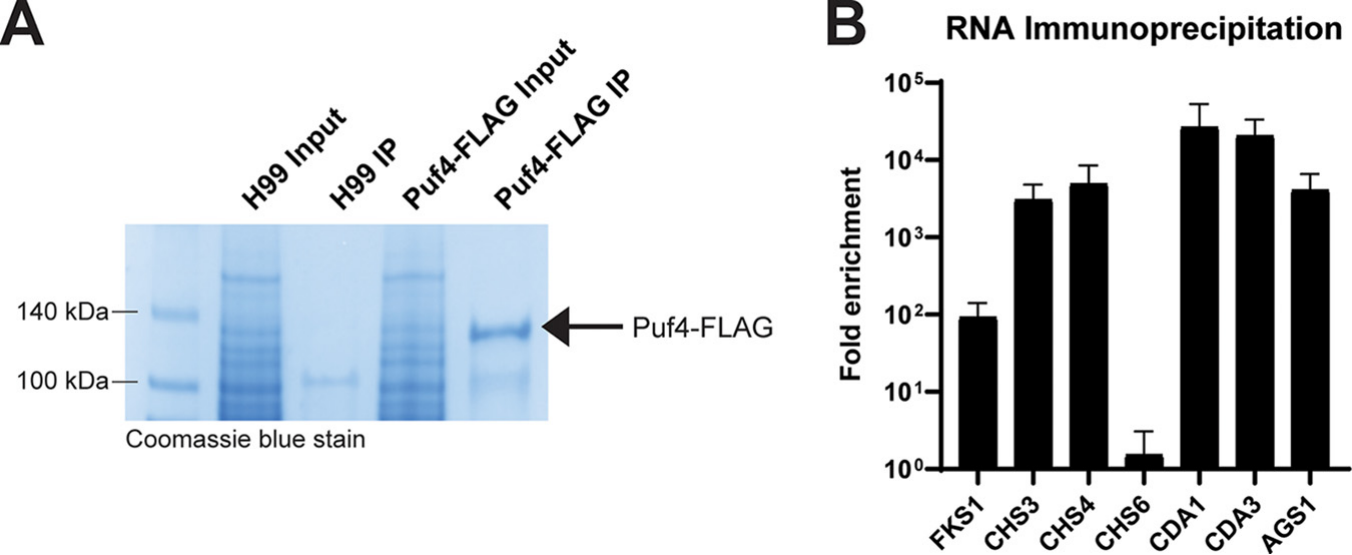
Puf4 binds to the cell wall biosynthesis mRNAs other than *FKS1*. Puf4 binding to cell wall biosynthesis mRNAs is determined by performing RNA immunoprecipitation (RIP). (A) A representative Coomassie blue-stained SDS-PAGE image is shown to confirm the enrichment of Puf4 in the IP eluate. Arrow indicates the band corresponding to Puf4-FLAG. A mock IP using wild-type cell lysate was included as a negative control. (B) Puf4 interacts with *CHS3*, *CHS4*, *CDA1*, *CDA3*, and *AGS1*. Fold enrichment is calculated relative to mock IP and normalized to the input using RT-qPCR. *CHS6*, a gene that does not contain PBEs, is included as a negative control for binding.

We first confirmed that Puf4 physically binds to the cell wall biosynthesis genes identified in our bioinformatics screen. To do so, we performed RNA immunoprecipitations (RIP) using the Puf4-FLAG cell line. We immunoprecipitated Puf4-FLAG using anti-FLAG antibody-coated beads and confirmed the enrichment of Puf4 in the elution by Coomassie blue stain (Fig. 5A, arrow indicates Puf4-FLAG). Following immunoprecipitations, we extracted RNA and performed real-time quantitative PCR (RT-qPCR) to analyze the enrichment of cell wall biosynthesis mRNAs in the Puf4-FLAG RIP compared to a mock RIP using untagged wild-type cells. Results showed that Puf4 binds to some cell wall biosynthesis genes including *FKS1*, *CHS3*, *CHS4*, *CDA1*, *CDA3*, and *AGS1* (Fig. 5B). *CHS6* was included as a negative control for binding since it does not contain a PBE or any motif that resembles a PBE.

**FIG 6:**
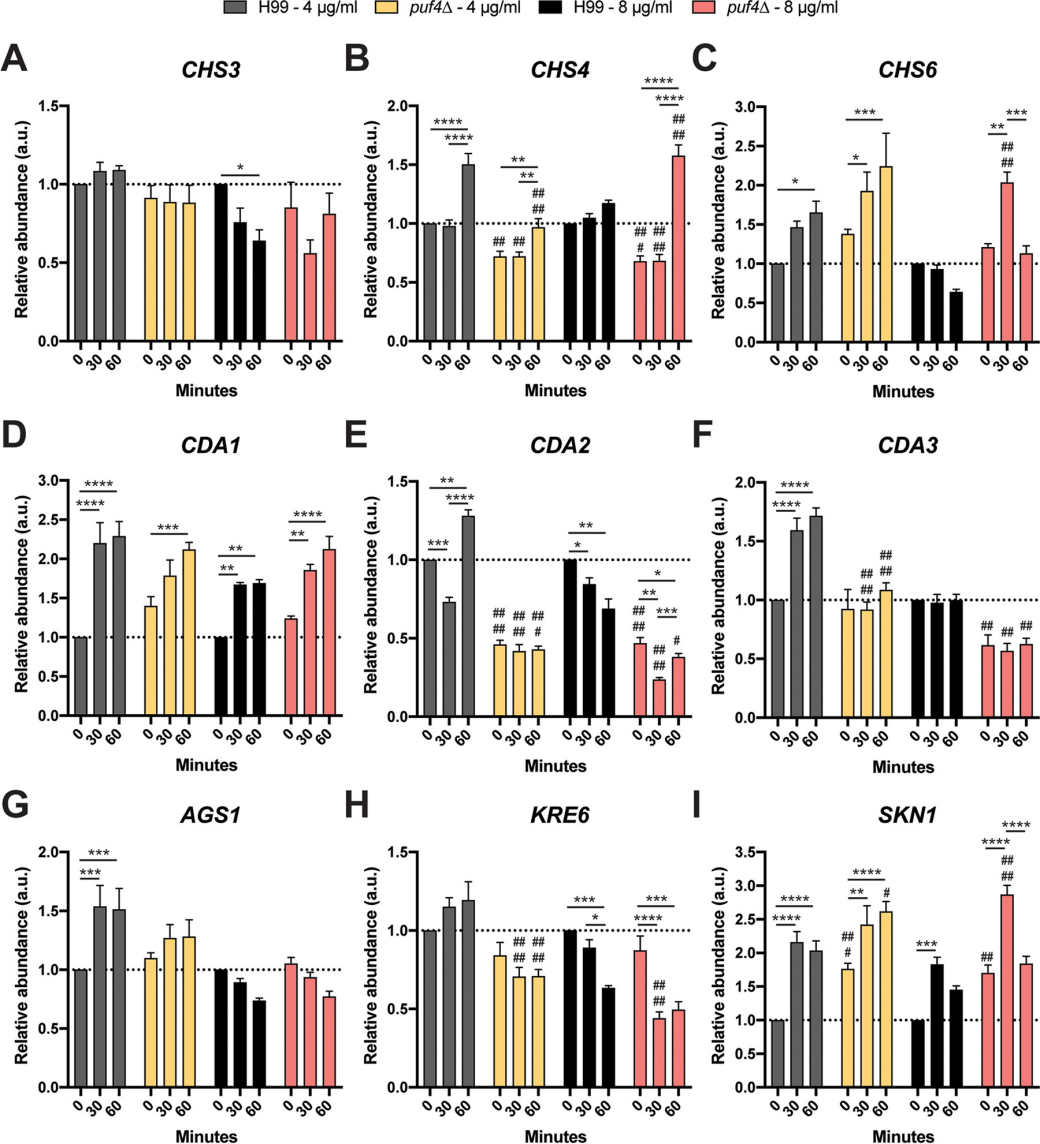
Deletion of *PUF4* leads to dysregulation of cell wall biosynthesis gene expression. The mRNA abundances of select cell wall biosynthesis genes were determined during a 60-minute caspofungin time course by collecting samples every 30 min and determining abundance by RT-qPCR using *GPD1* as a normalization gene. (A) *CHS3*; (B) *CHS4*; (C) *CHS6*; (D) *CDA1*; (E) *CDA2*; (F) *CDA3*; (G) *AGS1*; (H) *KRE6*; (I) *SKN1*. Three biological replicates with 2 technical replicates were plotted, and two-way ANOVA was used to determine statistical significance. ‘#’ denotes comparison between wild type and *puf4*Δ mutant, while ‘*’ denotes comparison between indicated time points within a strain. #/*, *P* < 0.05; ##/**, *P* < 0.01; ###/***, *P* < 0.001; ####/****, *P* < 0.0001.

We next asked if the caspofungin responsiveness of cell wall biosynthesis-related gene expression was dependent on Puf4. Following growth to the mid-log stage, we challenged both wild-type and *puf4*Δ cells with 4 or 8 µg/ml caspofungin and investigated the changes in the transcript abundance of genes involved in cell wall biosynthesis. We found that *CHS3* is significantly downregulated in the wild type following treatment with 8 µg/ml caspofungin for 60 min, whereas this reduction is absent in the *puf4*Δ cells (Fig. 6A). *CHS4* was downregulated in the *puf4*Δ cells. When cells were treated with 8 µg/ml caspofungin. *CHS4* was lower in the *puf4*Δ cells at steady-state levels and significantly upregulated in response to 8 µg/ml caspofungin in the *puf4*Δ cells (Fig. 6B). *CHS6* was upregulated in response to caspofungin at a greater magnitude in the *puf4*Δ cells than in wild type (Fig. 6C). Since Puf4 does not directly interact with *CHS6* (Fig. 5B), this change is likely orchestrated by a different effector protein. Analysis of the chitin deacetylase genes revealed that *CDA1* was upregulated in response to caspofungin, yet transcript abundance was not modulated by Puf4 (Fig. 6D). *CDA2* was downregulated in the *puf4*Δ cells and was responsive to caspofungin only when cells were treated with higher caspofungin concentrations (Fig. 6E). *CDA3* was downregulated in the *puf4*Δ mutant as well and did not change following caspofungin treatment (Fig. 6F). Elevated cell wall chitin content is shown to reduce susceptibility to caspofungin in *Candida* species (18). Therefore, altered expression of chitin synthase genes in the *puf4*Δ mutant may also contribute to the resistance phenotype in C. neoformans. The synthesis of chitosan from chitin is catalyzed by the chitin deacetylases, and chitosan is necessary for the integrity of the cell wall (30). Post-transcriptional regulation of mRNAs involved in chitin and chitosan synthesis might alter the cell wall composition.

Lastly, analysis of the α-glucan and β-glucan synthase genes revealed that AGS1 was not regulated by Puf4, KRE6 was downregulated in *puf4*Δ following caspofungin treatment, and SKN1 was upregulated in the *puf4*Δ mutant (Fig. 6G to I). Our quantitative analysis of the mRNAs involved in cell wall biosynthesis showed that Puf4 plays a regulatory role in the fate of these mRNAs and positively or negatively modulates their expression during caspofungin treatment.

### Puf4 stabilizes cell wall biosynthesis genes involved in chitin, chitosan, and **α**-glucan synthesis.

To gain more mechanistic insight on how Puf4 may control the cell wall biosynthesis-related transcript abundances, we investigated the mRNA stability of the same transcripts. mRNA stability is a crucial step in transcriptome and translatome remodeling to adapt to various environmental and compound stressors (31). Therefore, we hypothesized that Puf4 may modulate cell wall biosynthesis genes post-transcriptionally at the mRNA stability level.

**FIG 7:**
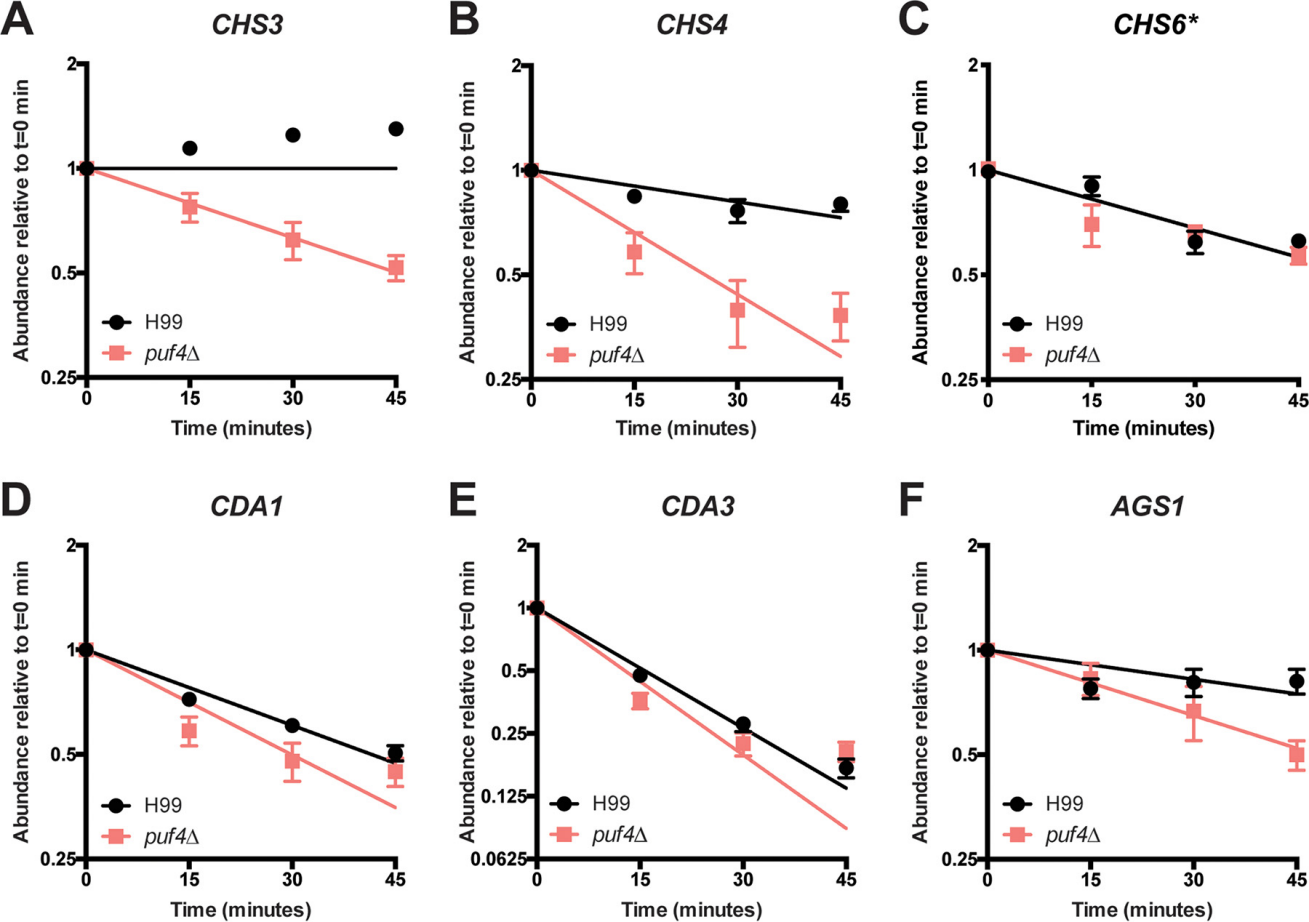
Puf4 stabilizes cell wall biosynthesis genes involved in chitin and α-glucan synthesis. *CHS3* (A), *CHS4* (B), *CHS6* (C), *CDA1* (D), *CDA3* (E), and *AGS1* (F) transcript abundances were determined using RT-qPCR following transcription shutoff to determine the mRNA decay kinetics. *GPD1* was utilized as the normalization gene. Fifteen minutes after transcription shutoff was denoted as *t* = 0. Three replicates were plotted, and differences between two strains were analyzed using one-phase exponential decay analysis.

We found that *CHS3* and *CHS4* were destabilized to a greater degree in the *puf4*Δ mutant, whereas *CDA1*, *CDA3*, and *AGS1* exhibited a slight reduction in stability in the absence of Puf4 (Fig. 7). Unlike other genes we investigated, *CHS6* does not contain a PBE and was included as a negative control. Not so surprisingly, *CHS6* stability was not altered in the *puf4*Δ mutant. mRNA stability data including the half-lives for each transcript are summarized in Table 2. Our results show that Puf4-mediated post-transcriptional gene regulation at the level of mRNA stability may be crucial for cell wall remodeling that contributes to the caspofungin resistance.

**TABLE 2:** mRNA half-lives of cell wall biosynthesis mRNAs in the wild-type and *puf4*Δ cells^*a*^

	Half-life (min)		*R*^2^		Standard error	
	H99	*puf4*Δ	H99	*puf4*Δ	H99	*puf4*Δ
FKS1	3.45562E+12	26.37	−0.009119	0.9195	0.001777	0.002064
CHS3	4.90492E+13	45.24	−1.521	0.7236	0.001651	0.001607
CHS4	99.28	25.29	0.449	0.406	0.0009544	0.003127
CHS6	59.06	49.44	0.7531	0.7657	0.001262	0.001409
CDA1	41.54	29.86	0.9176	0.7489	0.0007519	0.002262
CDA3	15.67	12.81	0.9761	0.9271	0.001634	0.004148
AGS1	107.5	47.96	0.09844	0.6275	0.001369	0.002002

a Half-lives were determined by performing one-phase exponential decay analysis.

### Extensive cell wall remodeling is a mechanism to resist caspofungin in the *puf4*Δ mutant.

**FIG 8:**
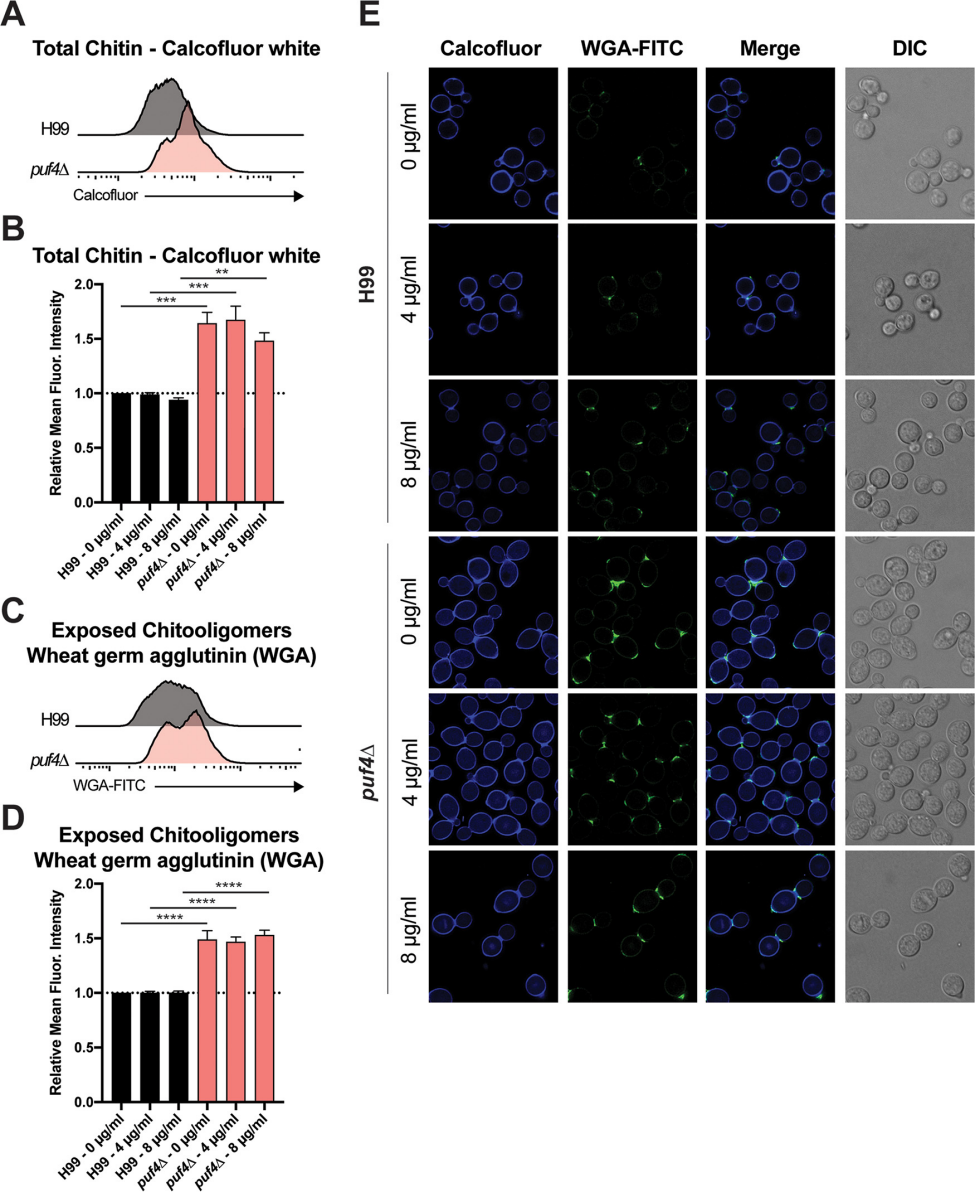
Deletion of *PUF4* leads to increased cell wall chitin and exposed chitooligomer levels. Cell wall chitin and exposed chitooligomer levels are increased in the *puf4*Δ mutant. Cells were grown to mid-log phase at 30°C and stained with calcofluor white and FITC-conjugated wheat germ agglutinin. Fluorescent intensity (A to D) and staining pattern (E) were determined using flow cytometry and fluorescence microscopy, respectively. For panels A to D, 3 biological replicates were plotted and one-way-ANOVA with Dunn’s multiple-comparison test was performed. *, *P* < 0.05; ***, *P* < 0.001; ****, *P* < 0.0001. DIC, differential interference contrast.

Since we have shown that Puf4 regulates cell wall biosynthesis-related transcript abundances, their mRNA stability, and translation in the case of *FKS1*, we further investigated the functional consequences of this Puf4 loss by assessing the abundances of various cell wall components using various dyes. We first analyzed the chitin and chitooligomer content using calcofluor and wheat germ agglutinin staining, respectively. Microscopy (Fig. 8E) and flow cytometry (Fig. 8A to D) analysis of the chitin content using the fluorescent dye calcofluor white showed that cell wall chitin and exposed chitooligomers are more abundant in the *puf4*Δ cells. In other pathogenic fungi such as *Candida* species and Aspergillus fumigatus, increased chitin content is protective against caspofungin (17, 18). Our data points to a similar mechanism in which cell wall composition more reliant on chitin might be key to the caspofungin resistance in the *puf4*Δ mutant.

**FIG 9:**
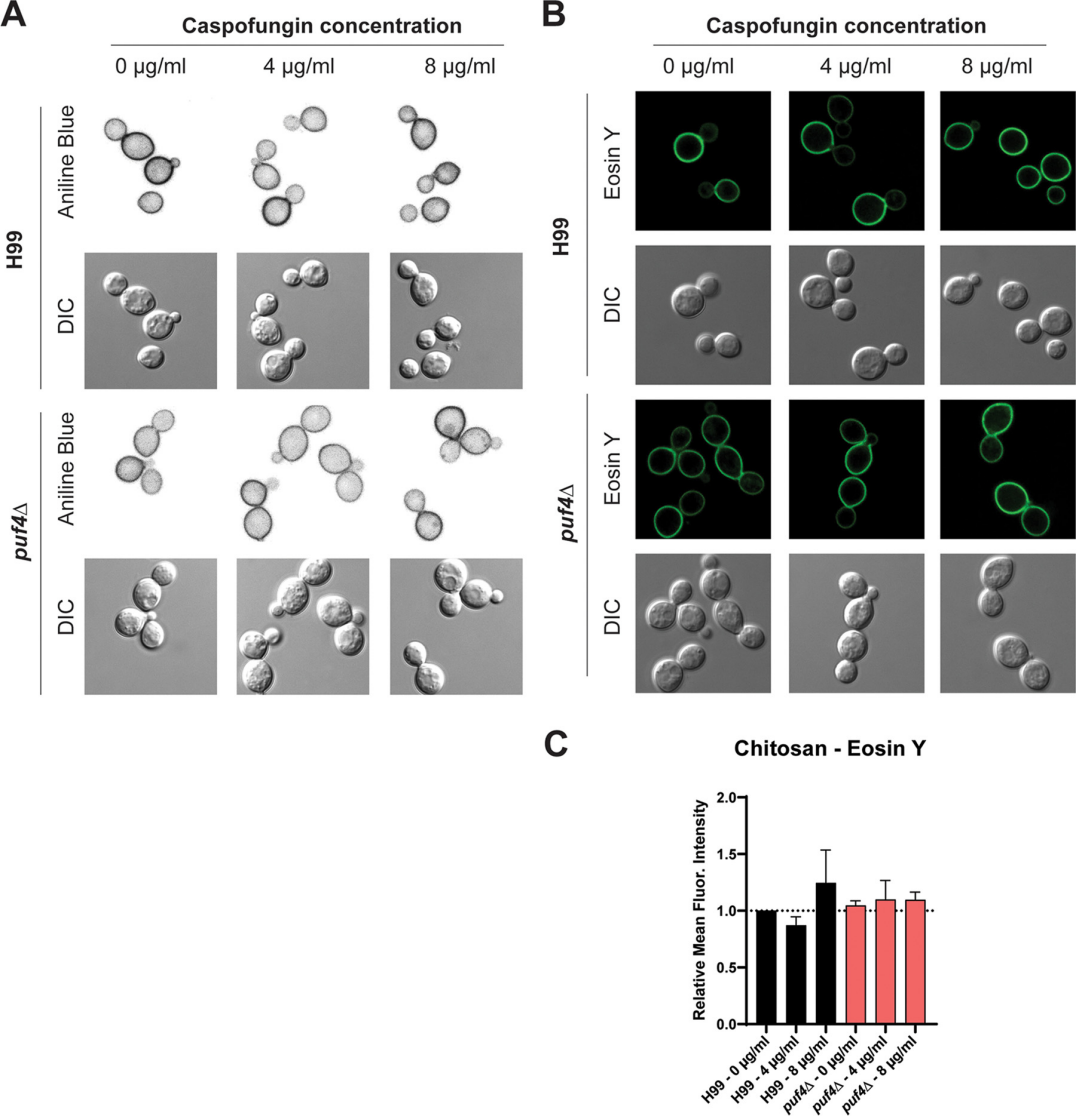
Puf4 regulates the cell wall β-1,3-glucan levels and does not regulate the cell wall chitosan levels. (A) The *puf4*Δ mutant has decreased levels of cell wall β-1,3-glucan. Cells were grown to mid-log phase at 30°C and stained with aniline blue to detect β-1,3-glucan. Representative aniline blue staining images for each strain are shown to assess both levels and localization. (B) Cell wall chitosan levels were quantified following eosin Y staining. Representative microscopy images are shown. (C) Mean fluorescent intensities of eosin Y-stained cells were analyzed using flow cytometry.

We then utilized aniline blue staining to investigate the β-1,3-glucan abundance in the cell wall of the wild-type and- *puf4*Δ cells. Aniline blue specifically binds to β-1,3-glucan (32). Following growth to mid-log phase, both wild type and the *puf4*Δ mutant were treated with 4 or 8 µg/ml caspofungin for 60 min and stained with aniline blue. Fluorescence microscopy revealed that aniline blue staining mainly localizes to the cell wall in the mid-logarithmic-stage cells as expected. Visual comparison of the aniline blue staining in the untreated wild-type cells to the *puf4*Δ cells revealed that cell wall β-1,3-glucan abundance is lower in the *puf4*Δ cells (Fig. 9A). Additionally, we observed that 8-µg/ml caspofungin treatment increases the aniline blue staining in the *puf4*Δ cells compared to untreated cells (Fig. 9A). This qualitative increase in the staining intensity is further supported by upregulation of the *FKS1* mRNA in the *puf4*Δ mutant following treatment with 8 µg/ml caspofungin as shown in Fig. 4A. Our attempts to quantify the aniline blue signal using flow cytometry were unsuccessful.

Lastly, since we have shown that chitin deacetylase genes were also regulated by Puf4 at the level of abundance and mRNA stability, and since they catalyze the synthesis of chitosan from chitin, we analyzed the cell wall chitosan levels. We stained the wild type and the *puf4*Δ mutant using eosin Y, which binds cell wall chitosan. Fluorescent signal was visualized using a confocal microscope and quantified using flow cytometry (Fig. 9B and C). Results revealed that even though cell wall chitin content is increased and chitin deacetylase genes are regulated, cell wall chitosan abundance was not altered in the *puf4*Δ mutant (Fig. 9).

In this study, we have demonstrated that the intrinsic caspofungin resistance of C. neoformans is regulated by Puf4 at the post-transcriptional level. This regulation is through the direct interaction of the mRNA encoding its target, *FKS1*, as well as through the regulation of multiple genes involved in cell wall biosynthesis. Post-transcriptional regulation of cell wall remodeling by Puf4 has functional consequences, as the absence of Puf4 results in massive remodeling of the C. neoformans cell wall components. Future work will investigate the effect of Puf4 on the translation of these target mRNAs, as well as the mechanism by which Puf4 itself is regulated.

## DISCUSSION

**FIG 10:**
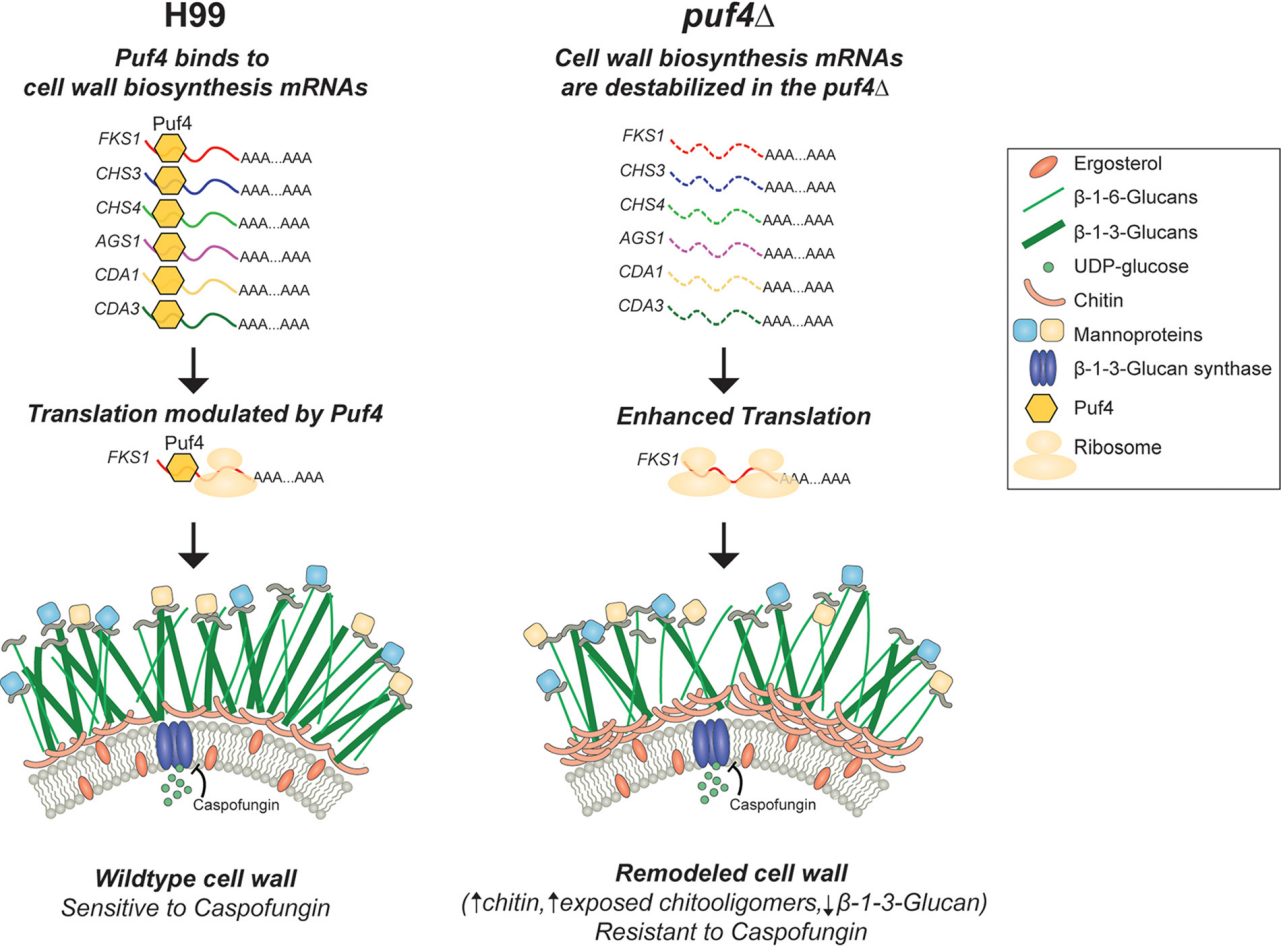
Model: post-transcriptional regulation of cell wall biosynthesis genes and cell wall remodeling by Puf4 is a path to caspofungin resistance in C. neoformans. In the wild-type cells, Puf4 controls the abundance and stability of cell wall biosynthesis genes including *FKS1*. This gene regulation has functional consequences in maintaining the cell wall composition and architecture. In the *puf4*Δ mutant, *FKS1* as well as other cell wall biosynthesis transcripts is destabilized. Additionally, *FKS1* mRNA is translationally more active in the *puf4*Δ mutant. Absence of Puf4-mediated gene regulation creates a cell wall that is richer in chitin and exposed chitooligomers while lacking in β-1,3-glucan. Post-transcriptional regulation of the cell wall homeostasis is the basis of caspofungin resistance in the *puf4*Δ mutant cells. Cell wall graphics are modified from reference 9 with permission of the publisher.

The search for novel antifungal therapies is an ongoing battle in medical mycology, especially with the growing number of fungal outbreaks and emerging drug resistance issues (20, 33). The latest class of antifungals approved by the FDA is the echinocandins, and *Cryptococcus* is intrinsically resistant to this class of antifungals (9, 14, 15, 34). Even though it is crucial to design new therapies, it is also imperative to understand the mechanisms of resistance to the existing antifungals to avoid similar scenarios and to design adjunctive therapies to remedy the current resistance issues. In this study, we elucidated the role of post-transcriptional gene regulation in the molecular mechanism of action behind caspofungin resistance. We discovered that Puf4, a pumilio domain-containing RNA binding protein, plays a role in the resistance phenotype by stabilizing the mRNAs encoding cell wall biosynthesis genes post-transcriptionally. The functional consequence of this interaction is a change in cell wall composition to a state that is more favorable during caspofungin challenge (Fig. 8). The intrinsic resistance of C. neoformans to caspofungin involves multiple signaling pathways (13, 20). For the first time, we have implicated post-transcriptional regulation of cell wall biosynthesis mRNAs, including the mRNA encoding the target of caspofungin, in this intrinsic resistance. Figure 10 shows a model of the path to caspofungin resistance.

PUF proteins, in addition to their interactions with other signaling proteins, alter mRNA function, and this is often secondary to the translational repression or the inhibition of mRNA decay (35, 36). Binding by Puf4 and other PUF proteins orchestrates mRNA fate-determining processes including stability, splicing, localization, and translatability (37, 38). For example, S. cerevisiae Puf4p stabilizes the transcripts involved in rRNA processing, and deletion of *PUF4* in S. cerevisiae causes defects in translation. Additionally, Puf4p plays a role in the recruitment of mRNAs to the translational machinery (39). In C. neoformans, we have shown that Puf4 is important in regulating mRNA stability of cell wall biosynthesis genes and the translation of *FKS1*.

In C. neoformans, Puf4 appears to play both positive and negative regulatory roles. Puf4 is a positive regulator of the unconventional splicing of the ER stress transcription factor *HXL1*. In contrast, Puf4 is a negative regulator of the *ALG7* mRNA, which is stabilized in the *puf4*Δ mutant (27). Interestingly, the Puf4 elements in the *HXL1* mRNA, like *FKS1*, are in the 5′ UTR, which may suggest that 5′ UTR Puf4-binding elements exert positive regulatory activity. Stabilization of the 5′ UTR Puf4 binding element containing *CHS3* and *AGS1* in the absence of Puf4 supports this claim (Table 1 and Fig. 7). The impact of Puf4 on its targets may be unique, and this may be due to the location of the PBE within the transcript, whether it is in the 3′ or 5′ UTR, introns, or exons, and may yield to variations in which Puf4 regulates transcript fate. Caspofungin challenge likely requires a reprogramming of gene regulatory networks for adaptation, and Puf4 and other related RNA-binding proteins may be involved in transforming the translating mRNA pool to best respond to the stress pharmacologically induced by caspofungin. The reduction of Puf4 protein levels in response to caspofungin treatment is consistent with the *puf4*Δ mutant being preadapted to caspofungin.

Cell wall maintenance and perturbations in response to drug-induced stress have a broad appreciation in medical mycology (40). For example, the *mar1*Δ mutant exhibits a defect in intracellular trafficking of cell wall synthases and therefore exhibits a cell wall composition that contains elevated exposed chitin and decreased glucan levels. These changes in the cell wall composition and exposure of different carbohydrates play meaningful roles in the immune recognition by the host and activate various signaling events in the host system (29). Another example is the enhanced recognition of the *ccr4*Δ mutant by alveolar macrophages due to increased unmasking of the β-1,3-glucan (41). The importance of Ccr4, an mRNA deadenylase, in glucan masking is further evidence that post-transcriptional processes are essential for adaptation to a number of stressors, including caspofungin treatment. In this study, we show that Puf4 is a major regulator of cell wall biogenesis. We report that cell wall biosynthesis genes showed different trends of expression in the *puf4*Δ mutant compared to wild type in caspofungin time course experiments. We have also shown that *FKS1*, *CHS3*, and *CHS4* mRNAs were destabilized in the absence of Puf4. This regulation of certain cell wall biosynthesis genes by Puf4 may be a necessary component of cell wall homeostasis under normal growth conditions as well as facilitating rapid changes in cell wall gene expression during adaptation to drug-induced stress. We have predicted that transcripts that have the PBE would have enhanced decay in the *puf4*Δ mutant, yet this was not true for all transcripts that carried PBEs. We investigated the stability of *CDA1* as a transcript that did not contain a completely canonical PBE, yet we observed a modest change in the mRNA stability. In that regard, it must be noted that *CDA1* contains a sequence motif that resembles a PBE (UGUAACGAUG) and binds Puf4. It is also likely that there are multiple RNA binding proteins regulating a single transcript.

The observation that *FKS1* mRNA is destabilized and yet Fks1 protein is increased in the *puf4*Δ mutant appears incongruous at face value. However, we know that mRNA decay and translation are interconnected processes from studies done in other eukaryotic systems. The translation rate of an mRNA can positively or negatively impact its stability (reviewed in reference 42). Even though there are different mechanisms in play, highly translated transcripts are often less stable. Many effector proteins are involved in Puf4-mediated gene regulation, and Puf proteins are known to disrupt translation and trigger or block mRNA decay. It is possible that in the absence of Puf4, *FKS1* is translated more efficiently, leading to its enhanced cotranslational degradation by cytoplasmic deadenylases and decapping enzymes (42).

The complex cell wall structure of C. neoformans protects the cell from extracellular stressors including antifungals. A sturdier cell wall can also serve as a less permeable barrier to antifungal drugs, making them less effective (43). We found that, following treatment with caspofungin, wild-type cells had an increase in the cell wall chitin. This increase was observed at the subpopulation level. This was an intriguing finding since subpopulation impact of antifungals is a neglected area of study, yet these heterogeneously resistant or tolerant populations may play important roles in antifungal resistance (44–46). Recent studies show that the effect of antifungals at subpopulation level is especially crucial to explain the growth of *Candida* species at supra-MICs (47). This is mainly due to slow growth of subpopulations compared to the rest of the cells. The heterogeneous nature of axenic microbial cultures, and life in general, is an intricate phenomenon that may significantly contribute to our understanding of antifungal resistance and tolerance. We hope that the single-cell genomics era will enhance our understanding of the antifungal resistance and tolerance pathways in more detail.

Puf4 is a bona fide downstream effector of the calcineurin pathway, as it was enriched in a phosphoproteomics screen of the *cna1*Δ mutant (24). Calcineurin was portrayed to be a longstanding player in the antifungal resistance of medically important fungi (48). Many groups have shown that disruption of the calcineurin pathway, genetically or pharmacologically, using novel or repurposed molecules, abolished the calcium homeostasis and led to death. This specific inhibition of calcineurin suggested to us that the calcineurin pathway, especially the downstream effectors, may contain potential targets which can be used as novel antifungal targets (49). Caspofungin treatment causes the translocation of a calcineurin-dependent transcription factor, Crz1. Translocation of Crz1 to the nucleus is an event that induces the transcriptional changes in gene expression in response to caspofungin. Yet, this transcriptional regulation does not contribute to the caspofungin resistance since the *crz1*Δ mutant exhibits a similar caspofungin sensitivity as that of wild-type cells (19). On the other hand, the absence of Puf4 causes a hyperresistant phenotype. While the absolute absence of Puf4 yields a resistance phenotype, we demonstrated that Puf4 protein levels drastically decrease following caspofungin treatment. Interestingly, this suggests that the downregulation of Puf4 may be necessary for the paradoxical resistance, hence the hyperresistant phenotype in the knockout. The converse was true for the overexpression of Puf4, which showed hypersensitivity to caspofungin. Designing a novel adjunctive therapy that can stabilize Puf4, or even better induce upregulation of Puf4, could be explored. If successful, this drug could be chemically tethered to caspofungin to achieve a dual goal.

Further work is needed to determine if the phosphorylation state of Puf4 is governed by calcineurin, or if there is another post-translational modification that is responsible for the rapid reduction in Puf4 protein abundance in response to caspofungin treatment. Inhibition of the mediator of Puf4 protein repression is another potential target to reverse the intrinsic resistance of C. neoformans to caspofungin. The post-transcriptional regulation of cell wall homeostasis by Puf4, a calcineurin-regulated RNA-binding protein, is another piece of the regulatory program that results in the intrinsic resistance. Screening additional RNA-binding proteins to understand the post-transcriptional regulatory network controlling cell wall dynamics, as well as investigating the regulatory connections with the cell integrity pathway and calcineurin signaling pathway, will further elucidate the response of C. neoformans to caspofungin that mitigates its toxicity and promotes intrinsic resistance. Further elucidation of this regulatory program may open new avenues to promote caspofungin sensitivity in C. neoformans through adjunctive therapy.

## MATERIALS AND METHODS

### Yeast strains and molecular cloning.

All strains used in this study were derived from Cryptococcus neoformans var. *grubii* strain H99, a fully virulent strain gifted by Peter Williamson (UIC, NIAID), which is derived from H99O gifted by John Perfect (Duke University). Primers used to build the knockout and FLAG-tagged complementation strains are included in Table S1 in the supplemental material. The plasmid construct to establish the PUF4-FLAG-(Hyg) mutant included native promoter and the terminator of the gene. The promoter and the coding sequence were amplified as a single fragment using a forward primer that contained a NotI cut site and a reverse primer that contained a SalI cut site as well as the FLAG sequence. The Puf4 terminator fragment was amplified using forward and reverse primers that contained SalI and BglII sites, respectively. Following restriction digest with respective enzymes, these fragments were cloned into pSL1180 containing the hygromycin B resistance cassette, as described previously (50). The construct containing PUF4-FLAG was introduced into the *puf4*Δ mutant using biolistics transformation. Copy numbers were determined using Southern blot analysis. A strain that is a single-copy-tagged complement and another strain that is a tagged overexpression strain were established. The FKS1-GFP cell line was a gift from Andrew Alspaugh, Duke University (29). The *puf4*Δ-FKS1-GFP cell line was generated by introducing the *puf4*Δ construct (27) into the FKS1-GFP parent cell line using biolistic transformation.

### Growth analysis: spot plates and plate reader assay.

Cells were grown overnight at 30°C in 5-ml cultures in yeast extract-peptone-dextrose (YPD) broth. Overnight cultures were washed with sterile distilled water, and the optical density at 600 nm (OD_600_) was made equal to 1 in water. Adjusted cultures were 1:10 serially diluted 5 times, and 5 µl of each dilution was spotted on YPD agar plates containing indicated concentrations of caspofungin (Sigma). Plates were incubated at 30°C for 3 days and photographed. For the kinetic plate reader assay, overnight cultures were washed with water once and then OD_600_ was made equal to 0.3 in YPD broth. Fifty microliters of YPD broth containing 2× the final caspofungin concentration was placed in each well, and then 50 µl of the OD_600_-adjusted cultures was placed in each well. The plate was incubated at 30°C for 20 h while shaking in a double orbital fashion, and OD_600_ was measured every 10 min during this kinetic assay.

### Electrophoretic mobility shift assay (EMSA).

EMSA reactions were set and analyzed as described previously (27). Briefly, all RNA binding reaction mixtures contained 5 µg of total protein lysate, 0.5 pmol of the TYE705-labeled oligonucleotide (IDT), and 4 µl 5× EMSA buffer (75 mM HEPES, pH 7.4, 200 mM KCl, 25 mM MgCl_2_, 25% glycerol, 5 mM dithiothreitol [DTT], and 0.5 mg/ml yeast tRNA) in a total volume of 20 µl. For competition reactions, 5×, 10×, and 20× more unlabeled wild-type or mutant oligonucleotides were added in addition to the TYE705-labeled oligonucleotide. Reaction mixtures were incubated at room temperature for 20 min, run on a DNA retardation gel, and then electrophoresed at 100 V. Gels were imaged using a LiCor Odyssey imaging system.

### Motif search-FIMO: find individual motif occurrences.

Cell wall biosynthesis genes were scanned for the Puf4-binding element using the FIMO tool on the MEME-suite version 5.1.0 (51). RNA sequences of the cell wall biosynthesis genes in Table 1 were acquired from FungiDB and provided as the input. The **UGUA**NNNN**UA** motif was scanned using the default settings. Only the given strand was searched, and the *P* value criterion was set as *P* < 0.0005 for significance cutoff. Results were manually curated to ensure accuracy in detecting the desired motif.

### RNA stability time course.

Overnight cultures grown at 30°C were used to inoculate 35 ml of YPD broth at an OD_600_ between 0.15 and 0.2 in baffled Erlenmeyer flasks. Cultures were grown in baffled flasks at 30°C while shaking at 250 rpm until they reached the mid-log stage—OD_600_ between 0.6 and 0.7. Mid-log-stage cultures were supplemented with 250 µg/ml of the transcriptional inhibitor 1,10-phenanthroline (Sigma). Then, 5-ml aliquots of each culture were transferred to snap-cap tubes and pelleted every 15 min for 60 min. Fifty microliters RLT buffer supplemented with 1% β-mercaptoethanol was added to each pellet prior to flash freezing in liquid nitrogen. Pellets were stored at −80°C until RNA extraction. Cells were lysed by bead beating using glass beads. RNA was extracted from each sample using the RNeasy minikit (Qiagen) following manufacturer’s instructions. RNA was DNase digested on-column using the RNase-free DNase kit (Qiagen) or using the Ambion Turbo DNA-free kit (ThermoFisher). cDNA for real-time quantitative PCR (RT-qPCR) was synthesized using the Applied Biosystems high-capacity cDNA reverse transcription kit (ThermoFisher) using random hexamers. Eight hundred to 1,000 ng RNA was used to synthesize cDNA. Samples were quantified using the second-derivative-maximum method and fitted to a standard curve of five 4-fold serial dilutions of cDNA. For experimental samples, cDNA was diluted 1:5 in nuclease-free water. To make the reaction mixture, 5 µl of the 2× SYBR green Blue Mix (PCR Biosystems) was combined with 4 µl of 1.5 µM primers (970 µl water + 15 µl forward + 15 µl reverse). Nine microliters of reaction mixture was placed in each well, and 1 µl of either experimental samples or standards was added to the respective wells. Samples from 3 biological samples in duplicate wells were tested. Primer sequences are listed in Table S1 along with the gene identifiers (IDs). Statistical differences were compared by determining the least-squares fit of one-phase exponential decay nonlinear regression analysis with GraphPad Prism software. Significance between curves was detected with the *P* value cutoff 0.05, which determined that the data from two different curves create different regression lines, therefore yielding to different half-lives of the same transcript investigated in different mutants.

### Caspofungin time course.

Cells were grown to the mid-log stage as described above. At this stage, cultures were supplemented with 4 or 8 µg/ml caspofungin, and 5-ml aliquots were collected in snap-cap tubes at 30 and 60 min. Fifty microliters of buffer RLT supplemented with 1% β-mercaptoethanol was added to each pellet prior to flash freezing in liquid nitrogen. Pellets were stored at −80°C until RNA extraction. Cells were lysed by bead beating using glass beads. RNA was extracted from each sample, cDNA was synthesized, and transcript abundances were calculated using RT-qPCR as described above. Primer sequences are listed in Table S1. Statistical differences were determined using two-way analysis of variance (ANOVA).

### Immunoblotting.

Cells were grown to the mid-logarithmic stage and treated with 4 or 8 µg/ml caspofungin for an hour. Cell pellets were flash frozen in liquid nitrogen and stored at −80°C. At the time of lysis, 50 µl cold lysis buffer (50 mM Tris HCl, pH 7.4, 150 mM NaCl, 1 mM EDTA, 1% Triton X-100, 10 µl/ml HALT protease, and phosphatase inhibitor [ThermoFisher]) was added, and cells were lysed by bead beating using glass beads. Two hundred fifty microliters of the cold lysis buffer was added to the beads, and lysate was extracted from the glass beads. Lysate was centrifuged at 20,000 × *g* for 15 min, and supernatant was transferred to a new tube. Protein quantities were measured using the Pierce 660-nm protein assay kit (ThermoFisher). Twenty-five micrograms of protein was run per sample on 4 to 15% Mini-Protean TGX stain-free precast gels (Bio-Rad) at 150 V. Total protein was imaged using a Bio-Rad gel documentation system with the stain-free gel setting. Gels were transferred to a nitrocellulose membrane and blocked with LiCor Odyssey blocking buffer for an hour. Then, they were incubated overnight with the mouse anti-FLAG antibody (1:1,000 in Tris-buffered saline–Tween 20 [TBS-T] with 10% LiCor Odyssey blocking buffer) at 4˚C. The blot was washed three times with 15 min incubations in TBS-T. Then, LiCor rabbit anti-mouse 800 secondary antibody (1:10,000 in TBS-T with 10% LiCor Odyssey blocking buffer) was added. The blot was incubated with the secondary antibody for an hour at room temperature and then washed with TBS-T, and the blot was imaged using a LiCor Odyssey imaging system.

### Polysome profiling coupled with RT-qPCR.

Polysome profiling was performed as described previously in reference 52. Briefly, cells were grown to mid-log stage at 30°C. Cycloheximide was added to the cultures at a 0.1-mg/ml concentration. Cells were washed with polysome lysis buffer (20 mM Tris-HCl, pH 8, 140 mM KCl, 5 mM MgCl_2_, 1% Triton X-100, 0.1 mg/ml cycloheximide), and pellets were stored at −80°C. Cells were lysed using bead beating in polysome lysis buffer for 5 min. Lysed cells were centrifuged at 21,000 × *g* for 5 min. Supernatant was transferred to a cold tube, and RNA in lysate was quantified using a NanoDrop spectrophotometer. Two hundred fifty micrograms RNA was layered on top of a 10 to 50% percent sucrose gradient and ultracentrifuged for 2 h at 39,000 rpm, 4°C. Following centrifugation, sucrose gradients were pushed through a flow cell using a peristaltic pump, and absorbance was measured at 254 nm to detect RNA. Sixteen-drop fractions were collected throughout the sucrose gradient, every other fraction was pooled, and RNA was precipitated by adding 3 volumes of 100% ice-cold ethanol. Samples were stored at −80°C for precipitation overnight. Samples were centrifuged at 20,000 × *g* for 10 min at 4°C. Ethanol was removed, and pellets were resuspended in 250 µl RNase-free water. RNA was further extracted by adding 750 µl of TRIzol LS (Invitrogen). Ribosome-associated RNA samples were DNase I treated, and cDNA was synthesized as described above. Distribution of *FKS1* mRNA throughout the gradient was analyzed using RT-qPCR.

### RNA immunoprecipitation.

RNA immunoprecipitation was performed as described previously in reference 53. Briefly, 2 liters YPD was inoculated with 5-ml overnight cultures. Cells were grown to an OD_600_ of 1.5 to 2 at 30°C in a shaking incubator. Each culture was divided, and 1 liter of cells was pelleted, flash frozen in liquid nitrogen, and stored at −80°C. Each 1-liter cell pellet was processed in tandem. Frozen cell pellets were ground using a coffee grinder for a total of 3 min and further ground using a mortar and pestle for 20 min. Frozen cell powder was resuspended in RIP buffer (25 mM HEPES-KOH, pH 7.9, 0.1 mM EDTA, 0.5 mM EGTA, 2 mM MgCl_2_, 20% glycerol, 0.1% Tween 20, 300 mM KCl, 1× Complete protease inhibitor [Roche], 50 U/ml RNaseOUT [Invitrogen]). Lysate was cleared by centrifugation at 27,000 × *g* for 40 min at 4°C. Cleared lysate was incubated with anti-FLAG antibody-coated magnetic agarose beads (Pierce) for 4 h at 4°C. Then, beads were magnetized and washed three times with RIP buffer. Protein was eluted using 50 µl of elution buffer (0.1 M glycine, pH 2). One eluate was analyzed by SDS-PAGE and stained with Coomassie blue. Other eluates were treated with 0.17 mg/ml proteinase K (Sigma) for 20 min at 37°C. Then, RNA was extracted using TRIzol. RNA was treated with DNase I, and cDNA was synthesized as described above. RT-qPCR was performed as described above using the primers in Table S1.

### Cell wall staining, microscopy, and flow cytometry.

Cells were grown to the mid-log stage and treated with caspofungin as described previously. Cells were prepared and cell wall components were stained for microscopic and flow cytometric analyses as previously published (29, 54). Briefly, cells were pelleted and washed with 1× PBS once. Cells were fixed with 3.7% formaldehyde for 5 min at room temperature and washed with 1× PBS twice.

Cells were stained with calcofluor and fluorescein isothiocyanate (FITC)-conjugated wheat germ agglutinin (WGA; Molecular Probes) to visualize chitin. Calcofluor dye stains the total chitin while WGA stains only the exposed chitooligomers. Cells were incubated in the dark with 100 µg/ml FITC-WGA for 35 min and then consecutively stained with 25 µg/ml calcofluor white for 15 min. Cells were washed twice with 1× PBS before analysis. Stained cells were imaged using a Leica TCS SP8 confocal microscope. For microscopy, WGA was detected using the GFP settings and calcofluor was detected using the 4′,6-diamidino-2-phenylindole (DAPI) settings. Images were taken using the 100× objective. Representative images were shown. Flow cytometry data were acquired using a BD LSRFortessa cell analyzer. WGA signal was detected using a 488-nm laser, and the calcofluor was detected using the 405-nm laser. Flow cytometry data were analyzed with FlowJo v10.0 software. Representative histogram graphs were shown.

Eosin Y staining was performed as described previously (30). Briefly, cells were grown as described above and treated with caspofungin. Cells were pelleted and washed with McIlvaine’s buffer (0.2 M Na_2_HPO_4_, 0.1 M citric acid, pH 6) 3 times. Pellets were resuspended in 500 µl McIlvaine’s buffer, and 30 µl of 5-mg/ml eosin Y was added to each tube. Cells were stained at room temperature for 10 min. Cells were washed once with McIlvaine’s buffer and resuspended in a 500-µl volume. Cells were analyzed on a microscope and flow cytometer using the FITC channel as described above.

Cells were stained with aniline blue to detect the β-1,3-glucan levels. Unfixed mid-log-stage cells in YPD, untreated and treated with caspofungin, were washed with 1× PBS and stained with 0.05% aniline blue (Wako Chemicals, Japan) for 10 min, and then cells were imaged using the DAPI channel on the Leica TCS SP8 confocal microscope. Representative images are shown.

### Statistical analysis.

Data analysis was performed using the GraphPad Prism software version 6. Each figure legend contains the statistical test information that is used to assess the statistical significance. Briefly, we have utilized the one-phase exponential decay analysis to determine the half-life of the mRNAs analyzed. Immunoblot data were analyzed using unpaired *t* test with Welch’s correction. Gene expression and microscopy quantification data were analyzed using either one-way or two-way-ANOVA followed by a *post hoc* multiple-comparison test. For all of the graphs, significance is shown as follows: *, *P* < 0.05; ***, *P* < 0.001; and ****, *P* < 0.0001. All error bars represent the SEM throughout the article.
