# Supplementary figures and images for: Puf4 Mediates Post-transcriptional Regulation of Cell Wall Biosynthesis and Caspofungin Resistance in Cryptococcus neoformans
Source: mBio. 2021 Jan 12;12(1):e03225-20. doi: 10.1128/mBio.03225-20 (PMC7844544; doi:10.1128/mBio.03225-20)

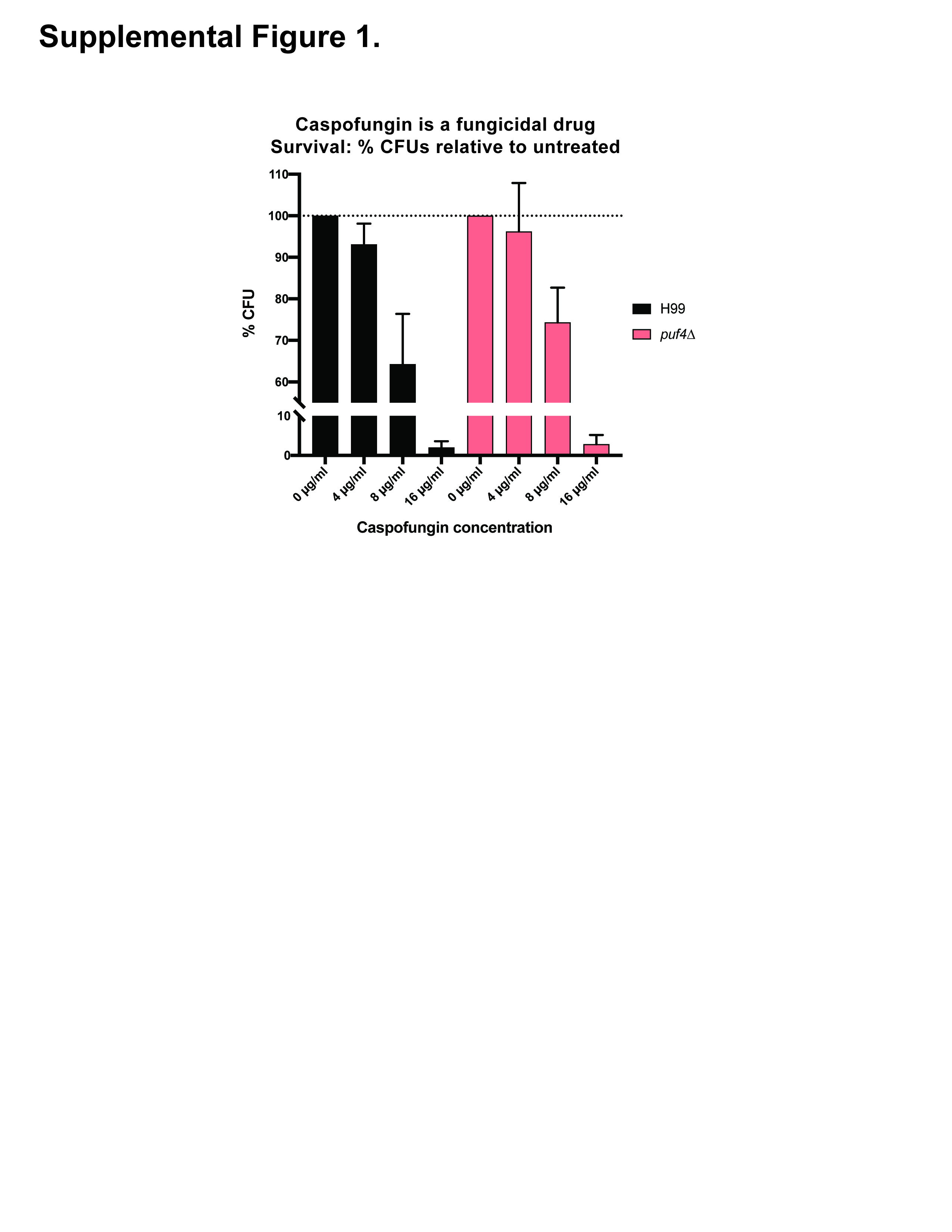

Supplement: FIG S1 [file mBio.03225-20-sf001.tif]

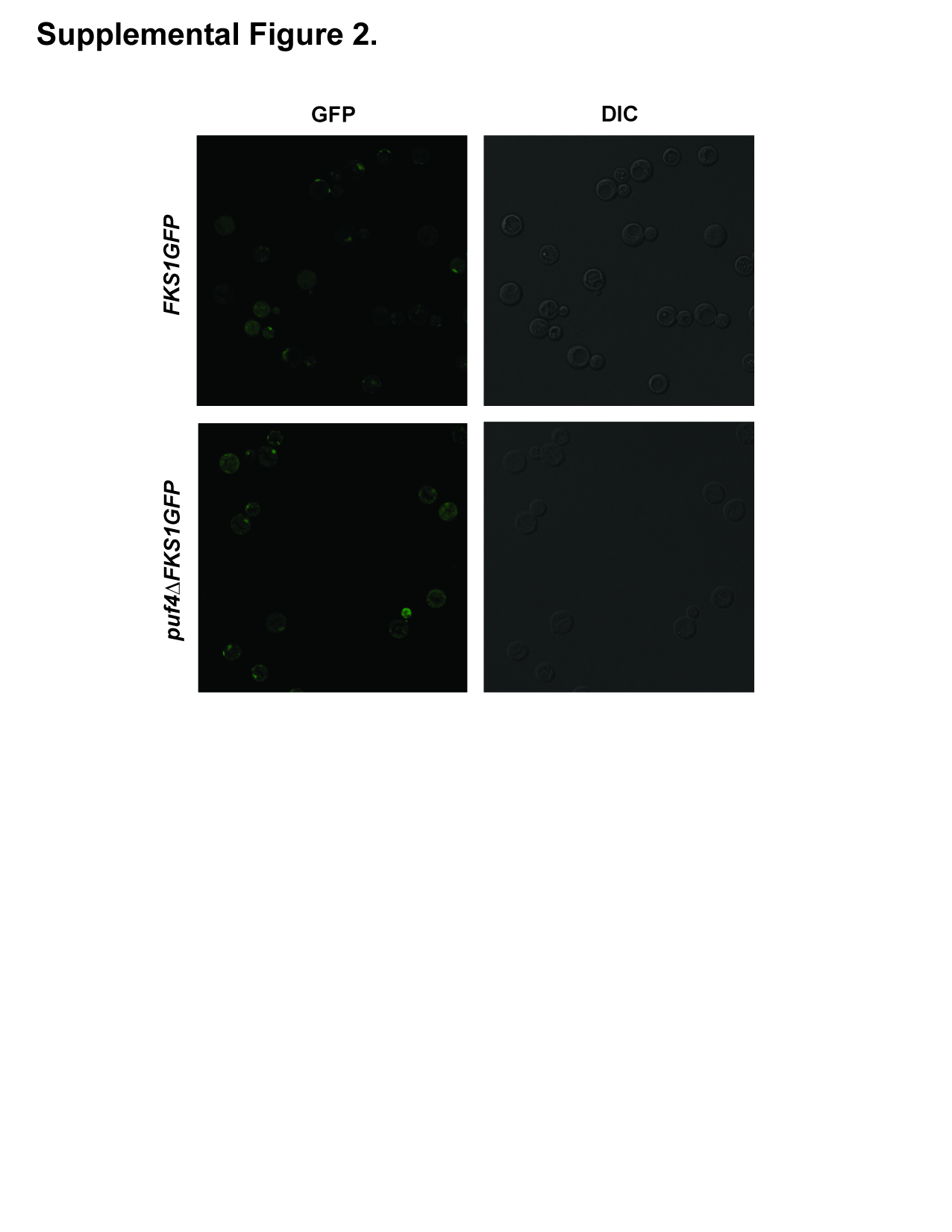

Supplement: FIG S2 [file mBio.03225-20-sf002.tif]
